# The Effect of Job Security on Deviant Behaviors in Diverse Employment Workplaces: From the Social Identity Perspective

**DOI:** 10.3390/ijerph18147374

**Published:** 2021-07-09

**Authors:** Chuanyan Qin, Kunjin Wu, Xiaolang Liu, Shanshi Liu, Wenzhu Lu

**Affiliations:** 1 School of Business Administration, South China University of Technology, Guangzhou 510641, China; qin_1994@126.com (C.Q.); bmssliu@scut.edu.cn (S.L.); wenzhuluscut@163.com (W.L.); 2 School of Business Administration, Guangdong University of Finance & Economics, Guangzhou 510320, China; 3 School of Management, Guangdong University of Technology, Guangzhou 510006, China; liuxiaolang0507@163.com

**Keywords:** job security, organizational identification, employment status, CWB, social identity perspective

## Abstract

Organizational scholars concur that job security can attach employees to a workplace and improve their job quality. The relationship between job security and employees’ deviant behaviors in the workplace, such as counterproductive work behavior (CWB), lacks insights into how or why this occurs, especially in a diversified employment context. To address these limitations, we developed a theoretical model of job security impact on employees’ CWB from the perspective of social identity. Analysis of employees (*N* = 208) and their supervisors in a China state-owned company were used to test the hypothesis. Results confirmed the negative relationship between job security and CWB; organizational identification partly mediates the relationship between job security and CWB. Moderated mediation analyses further indicate that the indirect effect of job security on CWB via organizational identification are stronger for temporary employees than for permanent employees. This article contributes to the understanding of job security’s impact on employees’ deviant behavior, practical implications and research aspects are discussed.

## 1. Introduction

Over the past two decades, the construct ‘job security’ has often been heard in discussions regarding the organizational behavior field. Job security is a critical element with respect to influencing employees’ psychological states and behaviors in the workplace [1]. A burgeoning body of job security research has shown the impact of job security on employees’ psychological state in workplace, including trust [2], anxiety [3], turnover intention [4], and expectation [5]. Numerous empirical studies have explored job security’s influence on employees’ job performance [5,6]. However, the link between job security and employees’ deviant behaviors in the workplace is yet to be fully explored. In this study, we will explore the relationship between job security and the classic negative behavior of CWB.

Existing research has investigated how job security affects employees’ psychological states and behavior from different perspectives, including resources conservation theory, social exchange theory, and job adaptation model [2,5,7]. However, most are based on employees’ mental or material resource exchange with external environmental, still lacking an understanding from the lens of employees’ internal thoughts about job security. We find an omitted but critical perspective—social identity [8]—can be used to deepen the understanding of how job security affect employees’ behavior. Social identification is the perception of oneness with or belongingness to some human aggregate, and it is also the same for organizations [8]. As suggested by a researcher, organizational identification means a self-conception in collective terms which will energize people to exert themselves on behalf of the group, facilitate the direction of efforts toward collective (instead of individual) outcomes [9]. According to social identity theory, when employees have sufficient perception about job stability, they are more inclined to establish self-concept in the organization, which can help adjust their behavior in line with organizational norms and goals and reduce negative behaviors that will harm the organization [10]. Thus, we will examine the mediating effect of organizational identification in the relationship between job security and employees’ CWB.

We suggest that although temporary workers also perceive identification to organization [11], their inferior employment status may affect the formation and effect of their organizational identification. According to the self-categorization theory that originated from social identity theory, an individual will strengthen their identification to a certain group based on their actual situation [12]. Such subgroups that form based on characteristics in a group not only affect individuals’ identification to a group, but also affect the impact that is exerted by the identification to the group [13,14]. Research has also confirmed that compared with organizations, employees have higher identification to their team [15,16]. Even in the same organization and workplace, the categorization of permanent and temporary employees may lead to differences in the organizational identification triggered by job security, as well as the impact of organizational identification on CWB. Therefore, we propose that employment status plays a moderating role in the indirect effect of job security on CWB via organizational identification.

**Figure 1:**
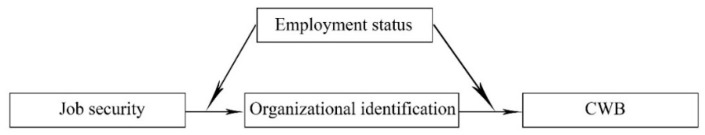
The hypothesized mediation and moderation model.

The aim of this article is to empirically test the relationship between job security and CWB, as well as the mediating mechanism, and boundary condition that influence this process. A conceptual model is shown in Figure 1. We expand the current literature as the following: first, we add to the research stream of job security by investigating its impact on employees’ deviant behavior (CWB). Second, using an alternative perspective—social identity—to elucidate job security influence on employees’ behavior, help uncover the mechanism through which job security takes effect. Third, we compare the temporary employees’ reactions to job security with permanent employees, demonstrating differences of psychological state and behavior between these two types of employees.

## 2. Theory Background and Hypothesis Development

### 2.1. Social Identity Perspective

Social identity related theories, which originated in the 1970s, are widely used in the organizational research field. The social identity theory pointed out that based on two psychological motivations of reducing uncertainty and self-improvement, individuals will try to obtain social identification from groups [17]. The identification to groups enables individuals to abide by the group norms, and act in accordance with the rules and preferences of the group [18]. Self-categorization theory, another branch of the social identity method, posits that individual identification generates in the process of distinguishing themselves from others [19], people establish identification based on the cognition of his/her roles, and then influence their outcomes in the group [18]. Subgroup research based on these two theories pointed out that although people have established the identification to a group based on the demand to reduce uncertainty and improve themselves, they still form subgroups based on their characteristics and attributes [20]. Additionally, they will strengthen the boundaries of this category to distinguish themselves from other subgroups. The formation of subgroups based on heterogeneity will lead to variations in individuals’ identification to the group [13].

In this study, we believe that when an employee is convinced that he/she belongs to an organization, they will identify with the organization, comply with the organization’s norms and goals, thereby reducing negative behaviors that may harm the organization. Meanwhile, the distinction of two subgroups (permanent and temporary workers) will make them feel differently about their belonging to the organization, which will affect formation and function of organizational identification. Therefore, based on the theory of social identity and self-categorization, this article discusses the impact of job security on employees’ CWB in hybrid employment context, as well as the role of organizational identification and employment status in this process.

### 2.2. Job Security and CWB

Job security, defined as ‘an employee’s subjective anticipation of employment stability and job continuity within an organization’ [1], is a predictor of employees’ behavior in a workplace [3,4,5]. It is worth noting that there exist differences between the construct of job security and job insecurity which also appears in research frequently. Although they look similar, the latter mainly underlines employees’ negative experiences due to the potential feeling of losing a job, including frustration, panic, etc. [21,22]. While, the former is concerned with the degree of satisfaction resulted from the stability and continuity of the job [1]. Previous studies demonstrated that job insecurity can trigger employees’ CWB via stimulating their negative emotions [23]. In contrast, we believe that job security can alleviate employees’ CWB in some way for the following reasons.

First, the relationship between employees and organizations is a kind of social exchange [24], in which organizations require employees’ help to achieve goals, and employees need organizations to provide economic and non-economic rewards. The principle of reciprocity also holds that when employees perceive the help from the organization, they will improve their behavior as return for the organization [25]. Therefore, we expect that when the organization offers employees the support of job stability and continuity, these employees will generate an obligation of giving back to the organization, including reducing CWB that will harm the organization. On the contrary, when it is felt that the organization does not provide sufficient job security, employees will not restrain themselves from engaging in negative behaviors. 

Second, a positive and stable working environment is one of the important sources of psychological resources in organizations [26], especially in a Chinese context that pursue stability [27]. Drawing from resource reservation theory, when individuals’ psychological resources are sufficient, they have more energy to control their behavior to not deviate from group norms. However, when psychological resources are exhausted, an individual may replenish their own resources by damaging the organization or others [28]. For employees with a high level job security, they can maintain better emotion and job satisfaction at work, and are less inclined to exert damage to the organization. While, low stability of work will make employees feel anxious and angry [29], leading to their venting of negative emotions by hurting theorganization, such as engaging in entertainment during working hours. Hence, we propose that an increase of job security will alleviate employees’ CWB. Thus,

**Hypothesis** **1.** *Job security is negatively related to employees’ CWB in the workplace*.

### 2.3. Mediating Effect of Organizational Identification

Social identity theory explains how individuals establish self-concept from the identity of the collectives they belong to [17]. Organization, as part and parcel of society, offer a scene for people to form self-concept. Some scholars considered organizational identification as a cognitive construct, some as emotion construct, the most persuasive definition, which contains both factors, is ‘perception of oneness with, or belongingness to, an organization where the individual defines him or herself in terms of the organization in which he or she is a member’ [30]. Based on the social identity theory, a stable working environment help employees feel that they belong to the organization, improve their perception of affiliation, which in turn affects their behavior in the workplace.

On one hand, job security provides a stable and controllable working environment for employees, increasing the opportunities for communication and connection between them with colleagues and supervisors, which is a critical antecedent of organizational identification [8]. Besides, high level job security will help create expectations of future work, making employees more motivated to establish links with organizations and deepen their self-concept organization. Both are important for employees to establish organizational identification [8,31]. Thus, we expect that the more closely organizations stick to a contract, the deeper employees’ identification to the organization will be. Empirical evidence also proved that high job security can strengthen employees’ organizational identification [32].

On the other hand, we predict that high organization identification can alleviate employees’ CWB. When employees perceive more identification and attachment to an organization, their psychology and behavior will make the same change accordingly. Specifically, employees who share values and goals with the organization will integrate their interest with the organization goal, prevent damage that may have happened in the organization, and follow with a drop in their deviant behavior [33]. On the contrary, if they do not have enough identification to the organization, they may pay more attention to own interests, rather than organization objective. Additionally, they will more likely damage the organization to satisfy their own needs, such as taking possession of the organization’s property, conflicting with others (both of which are part of CWB) [34,35]. Based on the reasoning above, we expect that organizational identification plays a role in the occurrence of CWB and that employees with a high level identification to the organization may demonstrate less CWB.

According to the above, organizational identification is expected to play a mediating role between job security and CWB. The following hypothesis is proposed:

**Hypothesis** **2.** *Organizational identification mediates the relationship between job security and CWB in the workplace*.

### 2.4. Moderating Effect of Employment Status

Employment status refers to whether the respondent are temporary or permanent employees [36]. Hybrid employment mode leads to great differences in working environments of two types of employees. Organizations hire temporary employees in order to reduce labor cost and gain flexibility, however, will not give good human capital investment and treatment to them [37]. Compared with permanent employees, temporary employees are always confronted with worse working environments, non-core and unimportant tasks, less training opportunities, a lower salary, and fewer promotion opportunities [38]. These will lead to a weaker psychological connection between temporary employees and the organization [39,40]. As a result, temporary employees generally intend to transform into a permanent employee or find a formal job in the external labor market [41].

Drawing on the theory of self-categorization, individuals will classify themselves into a certain group based on their attributes and roles and distinguish themselves from other groups [19]. There are also subgroups that are based on different characteristics in the organization, which will affect employee’s identification to the organization [13]. In a hybrid employment context, there is a significant discrepancy between permanent and temporary employees in their situations and mindset [42]. It leads to big differences in their identification to the organization, and subsequent behavior. Therefore, we argue that employment status moderates relationships between job security and organizational identification, and between organizational identification and employees’ CWB for the following reason.

Temporary employees are more sensitive to the variation in job security situation, which is conductive for establishing organizational identification. Specifically, as a result of employment status difference, permanent employees in state-owned enterprises will almost never lose their job [43]. While, temporary employees need to face problems of low job stability and continuity, which makes them more sensitive to information related to job security. Thus, permanent employees will not react violently when they feel an increase in job security since they are accustomed to the steady state of a job. For temporary employees with low job security, the stability provided by the organization is a great support, which will improve their identification to the organization to a large extent [44]. Therefore, we expect that temporary employees are more sensitive to the variation of job security and organizational identification, and we proposed that:

**Hypothesis** **3a.** *Employment status moderates the positive relationship between job security and organizational identification, that for temporary employees, this relationship will be stronger*.

Consistent with our discussion above, temporary employees’ groups are more sensitive to the support and investment from the organization, so they will make greater returns to the organization based on the increase of organizational identification, such as preventing themselves from conducting CWB. On the other hand, when the identification to organization increases, temporary employees will be more inclined to transform into permanent employee in the organization, that is, to change their employment status [41]. Driven by this motivation, temporary employees will improve their behavior in the organization to gain recognition from supervisors, so as to realize the transformation of employment status.

**Hypothesis** **3b.** *Employment status moderates the positive relationship between organizational identification and CWB, that for temporary employees, this relationship will be stronger*.

Based above, we supposed that when perceiving an increase in job security, temporary employees will experience a faster rise in their organizational identification, and further demonstrate a drop in CWB. Employment status can moderate the indirect relationship between job security and CWB through organizational identification. Hence, the hypothesis is as follows:

**Hypothesis** **4.** *Employment status will moderate the strength of the mediated relationships between job security with CWB via organizational identification, such that the relationships will be stronger for temporary employees than for permanent employees*.

## 3. Methods

### 3.1. Sample and Procedure

Data were collected from human resource management department, employees, and their corresponding supervisors in a Chinese state-owned company, which have three branches in Hunan, Xinjiang and Liaoning provinces. Participants come from nine sections, including flight, service, management, marketing, etc. We first asked human resources management department of headquarters to provide employees’ employment status and distribute questionnaires through online survey. A total of 300 questionnaires were issued to employees to measure their job security and organizational identification. One month later, human resources management issued questionnaires to 133 direct supervisors of these employees, to evaluate employees’ CWB, to reduce a common method of bias in this study. Finally, we deleted invalid samples and matching data of 208 employees and their 92 direct supervisors were obtained. We declared that the responses will be private at the beginning of the questionnaire.

Basic information of the sample is as follows: 109 were permanent employees (52.40%), 99 were temporary employees (42.31%). In total, 88 employees were male, and 120 were female (57.69%). As for the age, 98 employees were below 30 years old (47.12%), 77 were between 30 and 39 (37.02%), 32 were between 40 and 49 (15.38%), and only 1 was 50 years old or above (0.48%). In terms of educational status, 16 have received middle school education or below (7.69%), 34 have received high school or secondary school education (16.35%), 97 have a junior college’s degree (46.63%), 60 have a bachelor’s degree (28.85%), and 1 has a graduate degree (0.48%). In terms of tenure in this company, 13 have worked for less than 1 year (6.25%), 50 have worked for 1–3 years (24.04%), 28 have worked for 3–5 years (13.46%), 36 have worked for 5–7 years (17.31%), and 81 have worked for more than 7 years (38.94%). Among them, 65 were working in Hunan (31.25%), 34 were in Liaoning (16.35%), and 109 were in Xinjiang (52.40%).

### 3.2. Measure

For all measures, respondents rated the items on a 5-point scale, ranging from 1 = strongly disagree to 5 = strongly agree. The questionnaires were presented in Chinese language. Following previous guidance [45], we translated English scales into Chinese, and then back translated, to ensure the equivalence.

Job security. Job security was measured with a 6-items scale [46]. The original scale aimed at managers’ perceived job security, so we made an alteration to the objectives. Samples include “Employees in our firm can expect to stay for as long as they wish” and “Job security is almost guaranteed to employees in our firm”. The Cronbach α coefficient for the scale is 0.91.

Organizational identification. Organizational identification was measured by a 5-items questionnaire [47], which was rated by employees. Samples include “I feel strong ties with the company” and “I experience a strong sense of belonging to the company”. The Cronbach α coefficient for the scale is 0.84.

CWB. We adapted a 12-item scale to measure employees’ CWB to the organization [48]. Sample items include “Taken property from work without permission” and “Spent too much time fantasizing or daydreaming instead of working”. The Cronbach α coefficient for the scale is 0.87.

Employment status. Employment status was reported by human resource management department, temporary employees were coded as 0, and permanent employees were coded as 1.

Control variables. This paper controlled for several variables. Employees reported their gender (coded 1 = male; 2 = female), age (coded 1 = 29 or below; 2 = between 30 and 39; 3 = between 40 and 49; 4 = 50 and above), education experience (coded 1 = middle school or below; 2 = high school or secondary school; 3 = junior college; 4 = university; 5 = postgraduate or above), and tenure (coded 1 = 1 year or below; 2 = between 2 and 3 years; 3 = between 4 and 5 years; 4 = between 6 and 7 years; 5 = 7 years and above). Human resource management department reported employees’ working city (coded 1 = Hunan; 2 = Liaoning; 3 = Xinjiang).

### 3.3. Analyses

We first examined the discriminant validity of the focal scales with confirmatory factor analysis (CFA). Next, to test the hypotheses 1, 2, 3a and 3b, OLS regression analyses were conducted by using SPSS 22.0 (IBM Corp, Armonk, NY, USA). We entered control variables, including employees’ gender, age, education level, tenure, and working city, to account for autoregressiveness in these variables. When the interaction terms were significant (*p* < 0.05), we then plotted the interaction. Moreover, since job security and organizational identification were collected in one point of time, we launched a bootstrapping procedure (model 4, bootstrapping 5000 samples) to reexamine the indirect effect from job security to CWB via organizational identification (hypothesis 2), by using process macro [49]. Finally, we tested the moderated mediation effect (hypothesis 4) by bootstrapping 5000 samples to compute the bootstrap confidence intervals (model 58). Asymmetric bias-corrected 95% confidence intervals was used to evaluate the indirect effects [50].

## 4. Results

### 4.1. Confirmatory Factor Analysis

Before hypotheses testing, we conducted a confirmatory factor analysis (CFA) by AMOS 24.0 to test the construct validity of the major variables included in this study, namely, employment safety, organizational identification, and CWB. To improve fit indices, we performed model modification by adding covariance of the residuals, according to modification indices and theoretical justification [51]. The modified model yields a good fit: χ^2^(df) = 2.20, RMR = 0.057, CFI = 0.91, IFI = 0.91, and RMSEA = 0.076. The factor loadings of all indicators of their constructs ranging from 0.5 to 0.91, are significant at the 0.001 level. Compared to the 2-factor model that combined job security and organizational identification (χ^2^(df) = 3.10, RMR = 0.072, CFI = 0.84, IFI = 0.84, RMSEA = 0.101), and 1-factor model (χ^2^(df) = 4.56, RMR = 0.073, CFI = 0.73, IFI = 0.73, RMSEA = 0.131), the baseline model fits our data best.

### 4.2. Common Method Variance

As employees’ job security and organizational identification were reported by employees at the same time, we examined the potential common method variance using Harman’s one-factor test. Results show that the first factor explained only 32.52% of variance, seven factors explained 68.18% of the variation together, indicating that common method variance did not lead to big biases in our sample.

### 4.3. Descriptive Statistics and Correlations

According to independent samples *t*-test analysis results in Table 1, there were statistically significant differences between two types of employees in age (M = 1.42, SD = 1.94; t (206) = −5.269, *p* < 0.001), educational experience (M = 2.74, SD = 3.20; t (189.180) = −3.873, *p* < 0.001), tenure (M = 2.77, SD = 4.33; t (206) = −9.946, *p* < 0.001), job security (M = 3231, SD = 3.777; t (206) = −3.665, *p* < 0.001), organizational identification (M = 3.527, SD = 3.730; t (190.260) = −2.299, *p* < 0.05), and CWB (M = 2.565, SD = 2.106; t (180.940) =5.721, *p* < 0.001). These results support previous research and our argument that the aim of an organization’s adoption of hybrid employment is to save labor costs, and an organization will not make too much human capital investment, which can be seen from the lower educational level and shorter tenure of temporary employees. Correspondingly, temporary employees also show lower organizational identification and higher CWB than permanent employees. Therefore, it is of significance to explore how to affect the CWB of temporary workers and reduce it.

Table 2 displays correlations of the major variables and control variables. Both job security (r = −0.51, *p* < 0.01) and organizational identification (r = −0.62, *p* < 0.01) were significantly and positively correlated with CWB, and job security is positively related with organizational identification (r = 0.44, *p* < 0.01). Moreover, the strength of correlations among job security, organizational identification, and CWB all exhibit differences between two types of employment status.

### 4.4. Hypothesis Testing

We first tested direct effects of job security on CWB, and indirect effects through organizational identification. Hypothesis 1 predicted that job security is related to CWB. As Table 3 shows, we found the correlation between job security and CWB is significant (β = −0.48, *p* < 0.001), implying the association between job security and CWB is significant. Hence, hypothesis 1 is supported.

Hypothesis 2 predicted organizational identification mediating the relationship between job security and CWB. The results show that both job security (β = 0.43, *p* < 0.001) and CWB (β = −0.48, *p* < 0.001) were significantly associated with organizational identification, respectively; organizational identification was also negatively related with CWB (β = −0.52, *p* < 0.001). After taking these variables into account together, the coefficient between job security and CWB declined (β = −0.26, *p* < 0.001). Furthermore, mediation testing result by bootstrapping 5000 samples showed that both the direct effect of job security on CWB (b = −0.14, S. E. = 0.03, 95% confidence intervals = [−0.20, −0.08]), and the indirect effect via organizational identification were significant (b = −0.13, S. E. = 0.04, 95% confidence intervals = [−0.21, −0.07]), demonstrating that organizational identification plays a partly mediating effect in the relationship between job security and CWB. Thus, hypothesis 2 is supported.

Hypothesis 3a predicted that employment type will moderate the relationship between job security and organizational identification. Table 4 demonstrates that the interaction of job security and employment type is negatively related to organizational identification (β = −0.35, *p* < 0.001), which supports hypothesis 3a. This further indicates that for permanent employees, the positive relationship between job security and organizational identification is weaker than for temporary employees.

**Figure 2:**
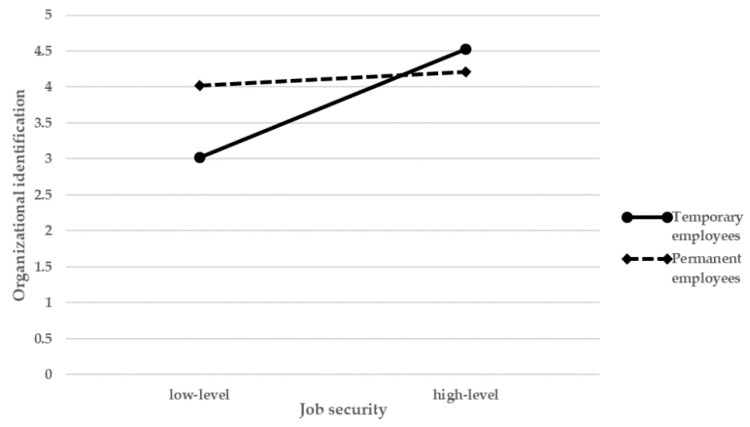
Interaction between job security and employment status on organization identification.

**Figure 3:**
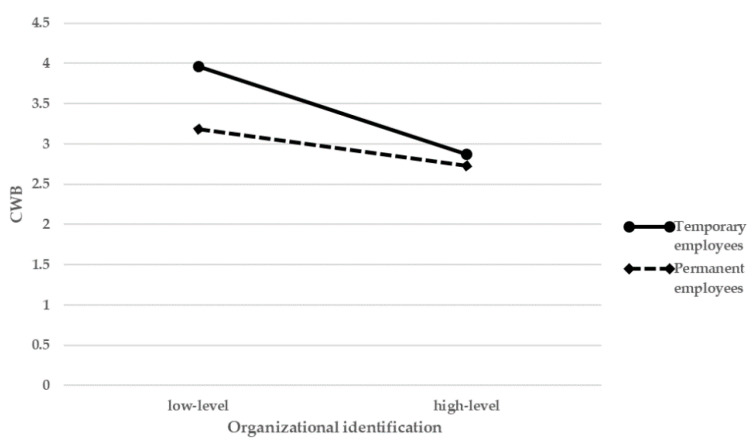
Interaction between organization identification and employment status on CWB.

Hypothesis 3b predicted that employment type moderates the relationship between organizational identification and CWB. As seen in model 4, the interaction of organizational identification and employment type is positively related to CWB (β = 0.17, *p* < 0.01), which provided support for hypothesis 3b. The result also means that negative reaction to organizational identification in permanent employees is gentler than temporary employees. The moderating effect of employment type is shown in Figure 2 and Figure 3.

Hypothesis 4 pertained to the moderated mediation of employment status of job security on CWB via organizational identification. Process macro was used to test the moderating effect. As seen in Table 5, the moderated mediating effect is significant (b = 0.22, S. E. = 0.08, 95% confidence intervals = [0.08, 0.41]). Besides, we tested the conditional effect at 1 SD above and below the mean and the result showed that for temporary employees, the indirect effect of job security on CWB is significant (b = −0.25, S. E. = 0.06, 95% confidence intervals = [−0.42, −0.18]), while not significant for permanent employees (b = −0.03, S. E. = 0.05, 95% confidence intervals = [−0.15, 0.01]). Thus, hypothesis 4 is supported.

## 5. Discussion

In this article, we tested the role of organizational identification as a mediator, employment status as a boundary condition to extend our understanding of how job security influences employees’ CWB. The results show that job security is negatively related to employees’ CWB through organizational identification. Employment status moderated the relationship between job security and organizational identification, as well as the relationship between organizational identification and CWB. Results of moderated mediation analyses demonstrate that temporary employees are apt to demonstrate a sharper decrease in organizational identification in response to low job security than permanent employees; when perceiving a low organizational identification, temporary employees tend to exhibit more CWB.

Our results support hypothesis 1, that job security negatively predicted the CWB. Additionally, results provide support for hypothesis 2, that organizational identification acts as a cushion between low job security and CWB, which is consistent with previous research [52]. Previous research explored the impact of job security on employees’ attitude and behavior, mainly from the perspective of psychosocial material resources exchange [5,7,53]. By focusing on employees’ inner cognition and thought about their group, we find that job security can change employees’ perceptions to an organization, and thus affecting their behavior, which provides a new perspective to understand effects of job security. Specifically, employees who perceive a continuous and stable working state are more likely to be inspired to establish self-concept in an organization, foster an identification to it, in turn helping employees change their behavior in the workplace, such as reduce deviant behavior, to protect the interests of the organization.

This article answers the call of research for more work on job security in diversified employment [37] and finds employment status is a key boundary condition in the process of job security effects. The results revealed that job security may elicit more pronounced psychological reaction for temporary employees than permanent employees, this result is in line with those of previous studies that employees of different employment status showed great differences in their psychological states and behavior [37,39,54]. More specifically, for temporary employees, the negative relationship between job security and organizational identification are stronger than permanent employees. Additionally, when an increase in job security is felt, temporary employees will experience a bigger enhancement in organizational identification. The explanation might be that for temporary employees who are in an embarrassing position [37], once the likelihood to be more stable than ever is felt, they will have a stronger perception of this support from the organization, and promote the sense of belonging to the organization, which is the basis of organizational identification [8].

The results also show that the negative relationship between organizational identification and CWB is stronger for temporary employees than for permanent employees. This phenomenon may due to the fact that when temporary employees have a higher organizational identification, they may be more inclined to transform into a formal member of the organization. To achieve this goal, they will prevent themselves from conducting negative behavior. In addition, the avoidance to risk makes temporary employees more sensitive to the level of job security [55], and more inclined to become a permanent employee in the organization. Therefore, Chinese cultural background of value stability also amplifies the moderating effect of employment status in the indirect relationship of job security and CWB via organizational identification [27].

One unanticipated finding is that, as shown in Figure 2, temporary employees perceived more organizational identification than permanent employees when the job security is at a high level. The reason might be that under a continuous and stable working environment, when supervisors match temporary employees’ preferences for work arrangement, working times, they will have an intense purpose to stay [56], and reinforce their identification to the organization.

### 5.1. Theoretical Implications

The results provide us with several theoretical implications. First, our research contributes to the literature on the relationship between job security and employees’ negative behaviors. Previous studies focused on the influence of job security on employees’ positive behavior [5,6]; whether job security can reduce employees’ negative behavior in workplace is still unknown. Although there are studies of CWB related to job insecurity [23], the constructs of job insecurity and job security exist substantial differences [1,21], so their influence on employees’ negative behavior should not be confused. Thus, this research shed light on the negative effect of job security on employees’ CWB.

Second, this study also makes an important theoretical contribution by further explaining how employees’ perceptions of the job security reduce CWB. Most existing research has focused on pressure control and social exchange perspective to understand job security’s impact on employees’ attitude and behavior [7,53,57], the current study uncovered a relatively overlooked theory of social identity, offering an internal perspective on how job security curbs employees’ CWB. We detail that a continuous and stable working state can help employees form self-concept in an organization, and in turn motivate them to integrate into a collective and alleviate acts of sabotage to the organization [8]. Hence, we highlight the utility of organizational identification in job security impact.

Finally, this research examined the boundary condition when job security decreases employees’ CWB via organizational identification, by accounting for the moderating role of employment status. Results showed that the relationship between job security and organizational identification, as well as between organizational identification and CWB is more sensitive for temporary employees. What is more, private enterprises differ, where permanent employees’ status is threatened by temporary employees [58], permanent employees in state-owned enterprises had a so-called ‘iron bowl’, and naturally take job security for granted on account of institutional reason [59]. This state-owned background also explains why the coefficient between job security and CWB (β = −0.48, *p* < 0.001) is greater than that of a study in Iran (β = −0.17, *p* < 0.001) [52]. Thus, we probed into the boundary conditions under which job security plays a role in such specific situation.

### 5.2. Practical Implications

The results suggest HR practitioners are supposed to notice that high job security may curb employees’ CWB by maintaining their organizational identification, that has several practical implications for supervisors in organizations. The first implication pertains to enhancing temporary employees’ perception of job security. Managers are supposed to convey the message that temporary employees will not be dismissed easily, to help them establish organizational identification, especially in the Chinese context that employees generally value employment security.

Second, different treatment of two types of employees in many aspects will lead to temporary workers engaging in more CWBs. Therefore, managers should maintain a certain degree of fairness in daily management and task distribution, to improve the psychological status and behavior of temporary employees.

Third, for permanent employees, job stability and continuity are more like a hygiene factor and are difficult to be used as an incentive role. Therefore, other measures should be applied to enhance the behavior of temporary employees, such as altering the compensation mechanism.

### 5.3. Limitations and Suggestions for Future Research

The generalizability of these results is subject to certain limitations that might be addressed in future research. First, although questionnaires of employees and supervisors are collected at different times, employees evaluate their job security and organizational identification simultaneously, which is not conducive to comprehend the causal relationship between them. Future research should measure these two variables at different times. The second limitation is that we collected data merely from a state-owned enterprise in China, which implies generalization of our results needs to be taken with caution; researchers in the future can address this limitation with more extensive sampling in various countries, industries, or in private enterprises. Third, our research only captured part of employees’ negative behavior, that is, CWB. It did not examine whether the job security instrumentally influenced more specific forms of negative workplace behavior, such as workplace rumors or aggressive behavior by employees, that occur often under unstable employment situations [60]. Future research should examine the effects of such variables on other types of performances. A strength of our design is that data were gathered from two sources: employees and their supervisors, which avoids appearance of a large common method bias.

## 6. Conclusions

Based on social identity perspective, the current study was designed to determine the effect of job security on CWB, the salient role that organizational identification played in the process, and the boundary conditions when job security impacts occur. Results showed that employees who perceived high job security are more likely to identify with their organization, and then reduce their CWB. Employment status moderated the negative effects of job security on organizational identification, and organizational identification on CWB, in which temporary employees demonstrated more intensely a reaction than permanent employees. This study offers some insight into how perception of job security affects employees’ CWB in the diversified employment situation.

## Figures and Tables

**Table 1:** Independent-samples *t*-test for employees’ demographic information.

		M	Mean	SD	t	df	Sig. (Two Tailed)
Gender	Temporary employees	99	1.53	0.502	−1.436	202.705	0.153
	Permanent employees	109	1.62	0.487			
Age	Temporary employees	99	1.42	0.608	−5.269	206	0.000
	Permanent employees	109	1.94	0.773			
Education	Temporary employees	99	2.74	0.943	−3.873	189.180	0.000
	Permanent employees	109	3.20	0.767			
Tenure	Temporary employees	99	2.77	1.168	−9.946	206	0.000
	Permanent employees	109	4.33	1.098			
JS	Temporary employees	99	3.231	1.118	−3.665	206	0.000
	Permanent employees	109	3.777	1.030			
OI	Temporary employees	99	3.527	0.694	−2.299	190.260	0.023
	Permanent employees	109	3.730	0.571			
CWB	Temporary employees	99	2.565	0.650	5.721	180.940	0.000
	Permanent employees	109	2.106	0.488			

Note. JS: job security; OI: organizational identification; CWB: counterproductive work behavior.

**Table 2:** Correlations among the study variables.

	1	2	3	4	5	6	7
Gender							
Age	−0.12						
Education	0.35 **	−0.01					
Tenure	0.10	0.49 **	0.11				
JS	0.02	0.15 *	0.01	0.14 *	(0.70)		
OI	−0.01	0.14 *	−0.06	0.02	0.44 **	(0.60)	
CWB	−0.13	−0.17 *	−0.17 *	−0.21 **	−0.51 **	−0.62 **	(0.43)

Note. *N* (temporary workers) = 99; *N* (permanent workers) = 109. Gender coded: 1 = male, 2 = female. JS: job security; OI: organizational identification; CWB: counterproductive work behavior. Average variance extracted values are listed in parentheses. * *p* < *0*.05, ** *p* < 0.01 (two tailed).

**Table 3:** Regression result for job security, organizational identification, and CWB.

	OI	CWB	
	Model 2	Model 3	Model 4
Gender	0.04	−0.07	−0.05
Age	0.12	−0.06	0.01
Education	−0.07	−0.13 *	−0.16 **
Tenure	−0.10	−0.09	−0.14 *
Area	0.01	−0.01	−0.00
JS	0.43 ***	−0.48 ***	−0.26 ***
OI			−0.52 ***
R^2^	0.19	0.28	0.50
F	8.85 ***	14.603 ***	30.60 ***

Note. N = 208. JS: job security; OI: organizational identification; ES: w = employment status; CWB: counterproductive work behavior. * *p* < 0.05; ** *p* < 0.01; *** *p* < 0.001 (two tailed).

**Table 4:** Moderating effects of employment status.

	OI		CWB	
	Model 1	Model 2	Model 3	Model 4
Gender	0.04	−0.04	−0.05	−0.04
Age	0.12	0.06	0.02	0.03
Education	−0.09	−0.11	−0.13 *	−0.12 *
Tenure	−0.17	−0.13	−0.05	−0.06
Area	−0.01	−0.02	0.02	0.01
JS	0.41 ***	0.66 ***	−0.24 ***	−0.20 ***
OI			−0.50 ***	−0.63 ***
ES	0.13	0.14	−0.18 **	−0.19 **
JS*ES		−0.35 ***		
OI*ES				0.17 **
R2	0.19	0.25	0.52	0.53
F	8.03 ***	9.55 ***	28.61 ***	26.90 ***

N = 208. JS: job security; OI: organizational identification; ES: w = employment status; CWB: counterproductive work behavior. * *p* < 0.05; ** *p* < 0.01; *** *p* < 0.001 (two tailed).

**Table 5:** Moderated mediation effects of job security on CWB via organizational identification.

	b	S.E.	LLCI	ULCI
Moderated mediation effect	0.22	0.08	0.08	0.41
−1 SD	−0.25	0.06	−0.42	−0.18
+1 SD	−0.03	0.05	−0.15	0.01

Note. N = 208. Results are based on 5000 bootstrap samples. LLCI = lower level 95% confidence interval; ULCI = upper level 95% confidence interval.

## Data Availability

The datasets used and analyzed in the current study are available from the corresponding author upon reasonable request.
